# Associations of dietary anthocyanidins intake with body composition in Chinese children: a cross-sectional study

**DOI:** 10.29219/fnr.v65.4428

**Published:** 2021-08-04

**Authors:** Gengdong Chen, Yan Li, Shujun Liang, Jinqiu Xiao, Xinyu Duan, Yuntao Zhou, Yanqing Zeng, Fanyiwen Sun, Shiksha Shrestha, Zheqing Zhang

**Affiliations:** 1 Foshan Institute of Fetal Medicine, Department of Obstetrics, Southern Medical University Affiliated Maternal & Child Health Hospital of Foshan, Foshan, China; 2 Department of Nutrition and Food Hygiene, Guangdong Provincial Key Laboratory of Tropical Disease Research, School of Public Health, Southern Medical University, Guangzhou, China

**Keywords:** anthocyanin, fat mass, lean mass, abdominal obesity, handgrip strength

## Abstract

**Background:**

Previous animal and *in vitro* studies indicated that anthocyanidins might contribute to the prevention of obesity, while epidemiological evidences were scarce and had not been conducted in children.

**Objective:**

We explored the associations between anthocyanidins and body composition in children.

**Design:**

A cross-sectional study involving 452 children aged 6–9 years in Guangzhou, China, was carried out. Dietary information was collected using a 79-items food frequency questionnaire. Fat mass (FM), lean mass (LM), and fat mass percentage (FMP) at multi-sites (whole body, trunk, limbs, android area, and gynoid area) were measured using a dual-energy X-ray scan. Abdominal obesity was defined as an age- and sex-specific abdominal FM ≥ 85th percentile. Handgrip strength was measured using a hydraulic hand dynamometer.

**Results:**

After adjusted for several potential covariates, higher dietary intake of anthocyanidin (per one standard deviation increase) was associated with a 0.013–0.223 kg increase of LM, a 0.024–0.134 kg decrease of FM, and a 0.63–0.76% decrease of FMP at multi-sites (*P* < 0.05). Results were similar and more pronounced for delphinidin and cyanidin, but less significant for peonidin. Higher dietary anthocyanidin intake (per standard deviation increase) was associated with a 41.0% (*OR*: 0.59, *95%CI*: 0.37, 0.94) decreased risk of abdominal obesity. However, no significant associations were observed between anthocyanidin and handgrip strengths.

**Conclusions:**

Higher dietary intake of anthocyanidin and its components tended to be associated with better body composition, but not handgrip strength, in Chinese children at early age.

## Popular Scientific Summary

- Higher dietary anthocyanidins intake was favorably associated with higher lean mass, lower fat mass, and fat mass percentage in children at early age.
- Higher dietary anthocyanidin and cyanidin intake was associated with lower risk of abdominal obesity.
- No significant associations were observed between anthocyanidins and handgrip strength.

Since the 1980s, the global prevalence of overweight and obesity in childhood has increased dramatically, and the rate of increase is greater in children than adults (1, 2). The status of adiposity was likely to retain from childhood to adulthood and lead to cardiovascular (3) or diabetic consequences (4). Recent evidence showed that detrimental precursor cardiovascular processes might happen in those with overweight or obesity even at early age (5). Scientists should come up with strategies to prevent further deterioration of the situation. Body mass index (BMI) is most widely used for evaluating overweight and obesity. However, it is short to assess fat distribution. More precise measurement of body composition is also needed in this field.

Body composition was influenced by both physical activity and nutrition (6, 7). Multiple dietary nutrients (e.g., protein, carbohydrate, calcium, and vitamin D) (6, 8, 9) were reported to affect body composition. Anthocyanidin is one of the important subfamilies of flavonoids, which mainly source from plant foods. Cyanidin, delphinidin, and peonidin are major compounds of anthocyanidin. Former evidence showed that dietary anthocyanidin or foods rich in anthocyanidin benefits several chronic diseases, like cancers of gastrointestinal tract (10), cardiovascular events (11), and diabetes (12). Oxidation and inflammation were two important mechanisms of obesity development (1). Anthocyanidins were reported to be with the capacities against oxidation by activating the nuclear factor erythroid 2-related factor-2 (Nrf2)/heme oxygenase-1 (HO-1) signaling pathway (13), and reduce inflammation by inhibiting nuclear factor-kappa B activation and increasing peroxisome proliferator activated receptor-γ (PPAR-γ) gene expression (14). Besides, anthocyanidins were able to inhibit lipogenesis through the activation of adenosine monophosphate-activated protein kinase (AMPK) pathway (15). With the evidences presented above, anthocyanidins were also hypothesized to be effective in obesity prevention and in improving the body composition. Supplements of foods rich in anthocyanidin improve adiposity conditions by lowering the levels of inflammatory factors (e.g., IL-6, MCP-1, CRP, and TNF-α) in several mice models, like C57BL/6 (16). Intervention of anthocyanidin-rich foods or fruits juices was also found to improve obese or metabolism status in randomized clinical trial studies of overweight or obese adults (17–19). Foods rich in anthocyanidin and other flavonoids contributed to weight maintenance in a large cohort of 1,24,086 US men and women followed for up to 24 years (20). High amount of anthocyanidin intake also contributed to better lipid profiles in a cross-sectional study of 1,393 Chinese women (21). However, to the best of our knowledge, the associations between anthocyanidin and body composition had not been studied in children yet. Whether anthocyanidin can generate early prevention of childhood obesity or not is still unknown.

We aimed to explore the associations of dietary anthocyanidin and its major compounds with body composition and handgrip strength in a cross-sectional study based on Chinese children at early age.

## Materials and methods

### Subjects

The study was based on a children population in Guangzhou, China. Detailed information of the recruitment was described before (22). To be precise, a total of 521 subjects responded from the invitation letters (315 from 1,394) or advertisements and referrals [206] and finally recruited during December 2015 and March 2017. Sixty-nine subjects were excluded for the following reasons: twins [12], preterm birth [25], relative medical condition [12], and core data unavailable [20]. Finally, a total of 452 children (197 girls and 255 boys) were included in the study as volunteers and received physical examination. A written consent was well explained and obtained from each subject through his or her parents or legal guardian. The study was conducted in accordance with the Declaration of Helsinki and was approved by the ethics committee of the School of Public Health at Sun Yat-sen University (No. 201549).

### Dietary information

Dietary information over the prior year was collected using a validated (23) food frequency questionnaire (FFQ, 79-items) through face-to-face interviews. For each item, the children were asked to answer their consumption frequencies (never, yearly, monthly, weekly, or daily) and the amount (with the help of their parents or legal guardians if necessary). Colorful photographs of foods in standard portion sizes with same reference objects were provided to better estimate the exact quantity of each food items. The Chinese Food Composition (24) was used for the calculation of mean consumption of daily intake of energy and protein. Dietary intake of anthocyanidin and its compounds was calculated using The Chinese Food Composition (25). All nutrients were adjusted for energy using residual method (26) to attenuate the influence of dietary energy intake. A total of 28 children completed two FFQs (FFQ1 and FFQ2) and 3-day dietary records with the help of their guardians over an interval of 12 months. The Spearman’s correlation coefficients between FFQ1 and FFQ2 (*r* = 0.385–0.604) were significant (at the criteria α < 0.05) for soy food, fruits, anthocyanidin, cyanidin, and peonidin and were significant (at the criteria α < 0.10) for vegetables (*r* = 0.348, *P* = 0.070) and delphinidin (*r* = 0.345, *P* = 0.072). The corresponding Spearman’s correlation coefficients between FFQ1 and 3-day dietary records were 0.438 for soy food (*P* = 0.020), 0.367 for vegetables (*P* = 0.055), and 0.348 for fruits (*P* = 0.069).

### Dual-energy X-ray absorptiometry (DXA) scans

Abdominal fat and lean mass were measured using a whole-body Dual-energy X-ray absorptiometry (DXA) scanner (Discovery W; Hologic Inc., Waltham, MA, USA) and analyzed by an experienced technician. Subjects wore only light clothing without metal or objects with high density, and then hold the standard posture under the guidance of the technician during the scan. FM, and LM at whole body, trunk, limbs, android area, and gynoid area sites were analyzed by the machine’s inbuilt software, and FMP was then calculated. For quality control, a spine phantom was used for daily correction before formal scans. The coefficients of variation between two consecutive measurements with repositioning among 35 randomly selected children on the same day were 0.77–5.67% for FM or LM at multi-sites. Abdominal obesity was defined as an age- and sex-specific abdominal FM ≥ 85th percentile according to the criteria reported previously (22).

### Handgrip strength measurement

Handgrip strength (accurate to 0.1 kg) of children was measured using a Jamar^®^ Plus+ Hand Dynamometer obtained from JAMAR^®^ Hydraulic Hand Dynamometer, Sammons Preston, Bolingbrook, IL, USA. Subjects performed the measurement twice using both hands as per instructed, and the largest handgrip strength was recorded. The coefficients of variation between repeated measures after a 30-min interval of 28 random selected subjects were 9.48% for the left hand and 8.19% for the right hand.

### Potential covariates

Anthropometric measurements were performed with subjects wearing light clothing and shoes-off in standing position. Height (accurate to 0.1 cm) was measured using a standard stadiometer, and weight (accurate to 0.1 kg) was measured using a Tanita MC-780A (Tanita Corporation, Tokyo, Japan). Other information of potential covariates was collected using a structured questionnaire through face-to-face interview with the attendance of both children and their guardians. Information of both children (e.g., age, birth information, physical activity, and use of supplements) and their parents (e.g., household income and education) was collected. Physical activity was noted by a continuous 3-day (two weekdays and one weekend day) record over the prior week (27), which investigated the daily physical activities that children were engaged in and time expenditure of each items (accurate to 15 min).

### Statistical analysis

We presented continuous variables as means and standard deviations (SD) and categorical variables as frequencies and percentages. Dietary intake of nutrients was adjusted for energy using the residual method. Linear regression models were operated for exploring the associations of dietary anthocyanidin and its compounds (per one *SD* increase) with body composition at multi-sites and handgrip strength. Logistic regression models were performed to explore the association of dietary anthocyanidins with abdominal obesity. Two different models were carried out for the adjustments in the linear regression analyses and logistic regression analyses, with model 1 as the univariate model and model 2 adjusted for the child’s age, sex, height, weight, delivery way, household income, parental education, physical activity, use of calcium and multi-vitamin supplements, dietary intake of energy, protein, fat, carbohydrate, cholesterol, calcium, and vitamin D. Variables were brought into the regression equations using the ‘Enter’ method. Subjects were divided into quartile groups according to their body composition. One-way ANOVA analyses were operated to compare the dietary intake of anthocyanidins by the quartile of the body composition, and the Bonferroni method was used for multiple comparisons between groups. Stratification analyses of different sex were also performed. All statistical analyses were performed using SPSS 21.0 (SPSS Inc., Chicago, IL), and a two-side *P*-value < 0.05 was considered as of statistical significance. An 84.4% power was achieved to detect a change in slope from zero under to 0.14 (slope between anthocyanin and whole body fat mass) when the SD of the exposure is 1.00, the SD of the residuals is 1.00. A sample of 452 observations achieve 92.2% power to detect the associations between dietary cyanidin (per SD increase) and abdominal obesity. The prevalence of abdominal obesity is 18.8%, related odds ratio is 0.59, and the R-squared value of dietary cyanidin with other covariates is 0.404 in our study. The power estimate analyzes were performed using PASS software version 11.0 (NCSS, LLC).

## Results

**Fig. 1:**
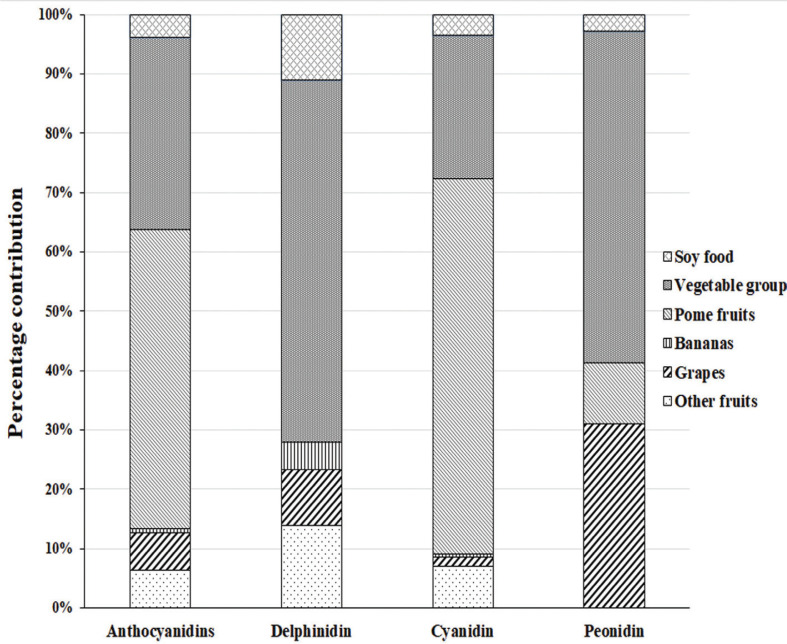
Foods contribution of anthocyanidins in the study. Total anthocyanidins were derived by summarizing delphinidin, cyanidin, and peonidin. Pome fruits included apple, pear, peach, pineapple, and plum.

A total of 197 girls (8.06 ± 0.96 years) and 255 boys (7.97 ± 0.91 years) were included in this study. Boys tended to be with higher BMI, weight, physical activities, and dietary intake of energy, protein, fat, carbohydrate, cholesterol, and calcium (all *P* < 0.05). No significant differences were observed for dietary anthocyanidin, its compounds, vitamin D, vegetables, fruits, and other variables between boys and girls (Table 1). Dietary anthocyanidin and its compounds were mainly obtained from pome fruits, vegetables, grapes, soy food, bananas, and other fruits (Figure 1). Subjects with higher fat mass percentage (FMP) at multi-sites exhibited a tendency of lower intake of total anthocyanidin, delphinidin, and cyanidin. Besides, subjects with higher trunk fat mass (FM) or android area FM tended to be with lower intake of total anthocyanidin, delphinidin, and cyanidin. No significant differences of dietary peonidin intake were observed among quartile groups of body composition at multi-sites (Supplementary Table 1). Dietary anthocyanidins were positively correlated with the dietary intake of carbohydrate (except for peonidin), calcium, vegetables, and fruits (*r’* = 0.102–0.781, all *P* < 0.05), but not for other nutrients, like protein, fat, cholesterol, and vitamin D (Supplementary Table 2).

**Table 1:** Characteristic of subjects included in the study

	Total (*N* = 452)	Girls (*N* = 197)	Boys (*N* = 255)	*P*
Age, years	8.01 ± 0.93	8.06 ± 0.96	7.97 ± 0.91	0.285
BMI, kg/m^2^	15.8 ± 2.75	15.2 ± 2.10	16.2 ± 3.09	**<0.001**
Height, m	1.29 ± 7.99	1.28 ± 7.81	1.29 ± 8.14	0.704
Weight, kg	26.4 ± 7.04	25.3 ± 5.51	27.3 ± 7.92	**0.003**
Physical activity, Met×h/d	39.9 ± 4.27	38.9 ± 3.87	40.6 ± 4.41	**<0.001**
Dietary energy intake, kcal/d	1,431 ± 434	1,336 ± 398	1,505 ± 449	**<0.001**
Dietary protein intake, g/d^a^	61.8 ± 9.11	58.8 ± 8.83	64.1 ± 8.65	**<0.001**
Dietary fat intake, g/d^a^	329 ± 69.7	316 ± 68.4	339 ± 69.1	**0.001**
Dietary carbohydrate intake, g/d^a^	193 ± 26.6	179 ± 22.3	204 ± 24.4	**<0.001**
Dietary cholesterol intake, mg/d^a^	348 ± 140	333 ± 121	360 ± 151	**0.041**
Dietary calcium intake, mg/d^a^	491 ± 140	475 ± 131	503 ± 145	**0.035**
Dietary vitamin D intake, IU/d^a^	90.4 ± 47.9	85.5 ± 45.7	94.1 ± 49.3	0.059
Dietary vegetable intake, g/d^a^	183 ± 94.3	188 ± 83.0	179 ± 102	0.339
Dietary fruit intake, g/d^a^	149 ± 99.2	148 ± 97.8	150 ± 100	0.449
Dietary anthocyanidin intake, mg/d^a^	6.88 ± 4.06	6.96 ± 4.05	6.81 ± 4.08	0.710
Dietary delphinidin intake, mg/d^a^	0.58 ± 0.44	0.60 ± 0.44	0.56 ± 0.43	0.257
Dietary cyanidin intake, mg/d^a^	5.36 ± 3.61	5.41 ± 3.55	5.32 ± 3.66	0.794
Dietary peonidin intake, mg/d^a^	0.94 ± 0.72	0.95 ± 0.69	0.94 ± 0.75	0.905
Delivery way, *N (%)*				0.068
Natural	228 (50.4)	109 (55.3)	119 (46.7)	
Cesarean	224 (49.6)	88 (44.7)	136 (53.3)	
Household income, Yuan×month^-1^, *N* (%)				0.913
≤ 15,000	217 (48.0)	94 (47.7)	123 (48.2)	
> 15,000	235 (52.0)	103 (52.3)	132 (51.8)	
Maternal education, *N* (%)				0.612
≤ 12 years	173 (38.3)	78 (39.6)	95 (37.3)	
> 12 years	279 (61.7)	119 (60.4)	160 (62.7)	
Paternal education				0.746
≤ 12 years	182 (40.3)	81 (41.1)	101 (39.6)	
> 12 years	270 (59.7)	116 (58.9)	154 (60.4)	
Use of calcium supplements, *N* (%)				0.192
No	269 (59.5)	124 (62.9)	145 (56.9)	
Yes	183 (40.5)	73 (37.1)	110 (43.1)	
Use of multi-vitamin supplements, *N* (%)				0.721
No	373 (82.5)	164 (83.2)	209 (82.0)	
Yes	79 (17.5)	33 (16.8)	46 (18.0)	

Continuous variables were presented as Mean ± standard deviation; Categorical variables were presented as frequency (percentage).

a , adjusted for energy using residual methods.

In univariate analysis, the higher dietary intake of anthocyanidin and its compounds (except for peonidin) was associated with lower FMP and lower FM (delphinidin, and cyanidin) at multi-sites (Supplementary Table 3). Results tended to be more pronounced after adjusted for potential covariates in Model 2 (Table 2). Higher dietary intake of anthocyanidin (per one *SD* increase) was associated with a 0.013–0.223 kg increase of LM, a 0.024–0.134 kg decrease of FM, and a 0.63–0.76% decrease of FMP at multi-sites (*P* < 0.05). Delphinidin (per one *SD* increase) was associated with a 0.057 to 0.168 kg increase of LM, a 0.033 to 0.114 kg decrease of FM, and a 0.49–0.97% decrease of FMP. Cyanidin (per one *SD* increase) was associated with a 0.014 to 0.209 kg increase of LM, a 0.058 to 0.114 kg decrease of FM, and a 0.54–0.70% decrease of FMP. Peonidin (per one *SD* increase) was only associated with a 0.031 kg decrease of gynoid area FM and a 0.46% decrease of gynoid area FMP. Results were similar, but generally less pronounced after stratified by sex (Supplementary Tables 4 & 5).

**Table 2:** Associations of dietary anthocyanidins with body composition after adjusted for potential covariates

	Anthocyanidin			Delphinidin			Cyanidin			Peonidin		
	*β*	*se*	*P*	*β*	*se*	*P*	*β*	*se*	*P*	*β*	*se*	*P*
Whole body												
Fat mass (FM), kg	−0.134	0.055	**0.015**	−0.114	0.053	**0.033**	−0.114	0.054	**0.037**	−0.079	0.051	0.124
Lean mass (LM), kg	0.223	0.053	**<0.001**	0.168	0.052	**0.001**	0.209	0.052	**<0.001**	0.059	0.050	0.237
Fat mass percentage (FMP), %	−0.628	0.195	**0.001**	−0.619	0.191	**0.001**	−0.540	0.194	**0.006**	−0.327	0.184	0.076
Trunk												
FM, kg	−0.064	0.027	**0.018**	−0.033	0.026	0.213	−0.058	0.027	**0.030**	−0.030	0.025	0.241
LM, kg	0.099	0.027	**<0.001**	0.057	0.027	**0.033**	0.093	0.027	**0.001**	0.034	0.025	0.180
FMP, %	−0.708	0.206	**0.001**	−0.485	0.191	**0.017**	−0.633	0.205	**0.002**	−0.366	0.194	0.060
Limbs												
FM, kg	−0.070	0.033	**0.036**	−0.079	0.032	**0.015**	−0.055	0.033	0.093	−0.049	0.031	0.117
LM, kg	0.121	0.031	**<0.001**	0.120	0.030	**<0.001**	0.110	0.030	**<0.001**	0.029	0.029	0.317
FMP, %	−0.749	0.269	**0.006**	−0.968	0.261	**<0.001**	−0.620	0.268	**0.021**	−0.387	0.253	0.127
Android area												
FM, kg	−0.009	0.005	0.055	−0.004	0.005	**0.413**	−0.008	0.005	**0.091**	−0.005	0.005	0.242
LM, kg	0.013	0.006	**0.033**	0.006	0.006	**0.285**	0.014	0.006	**0.017**	−0.004	0.006	0.506
FMP, %	−0.761	0.218	**0.001**	−0.511	0.214	**0.017**	−0.701	0.217	**0.001**	−0.302	0.206	0.142
Gynoid area												
FM, kg	−0.024	0.011	**0.027**	−0.033	0.011	**0.002**	−0.015	0.011	0.166	−0.031	0.010	**0.002**
LM, kg	0.034	0.012	**0.004**	0.014	0.012	0.235	0.038	0.012	**0.001**	−0.007	0.011	0.543
FMP, %	−0.755	0.242	**0.002**	−0.846	0.235	**<0.001**	−0.625	0.241	**0.010**	−0.463	0.227	**0.042**

Linear regression analysis, adjusted for covariates including: age, sex, height, weight, delivery way, household income, parental education, physical activity, use of calcium and multi-vitamin supplements, dietary intake of energy, protein, fat, carbohydrate, cholesterol, calcium, vitamin D.

**Table 3:** Associations of dietary anthocyanidins with abdominal obesity

Abdominal obesity	Per SD increase of dietary anthocyanidin and its main compounds											
	Anthocyanidin			Delphinidin			Cyanidin			Peonidin		
	*OR*	*95%CI*	*P*	*OR*	*95%CI*	*P*	*OR*	*95%CI*	*P*	*OR*	*95%CI*	*P*
Total (*N* = 452)												
Model 1	0.74	(0.56, 0.97)	**0.030**	0.81	(0.64, 1.07)	0.136	0.71	(0.53, 0.94)	0.016	1.05	(0.84, 1.32)	0.664
Model 2	0.59	(0.37, 0.94)	**0.028**	0.80	(0.50, 1.25)	0.323	0.56	(0.35, 0.90)	0.018	1.04	(0.74, 1.47)	0.802
Girls (*N* = 197)												
Model 1	0.80	(0.53, 1.22)	0.295	0.94	(0.64, 1.39)	0.757	0.78	(0.51, 1.19)	0.249	0.91	(0.68, 1.45)	0.961
Model 2	0.91	(0.45, 1.84)	0.790	1.27	(0.56, 2.88)	0.563	0.77	(0.37, 1.60)	0.489	1.60	(0.83, 3.08)	0.159
Boys (*N* = 255)												
Model 1	0.70	(0.49, 1.00)	0.052	0.72	(0.48, 1.06)	0.096	0.66	(0.45, 0.96)	**0.029**	1.09	(0.82, 1.45)	0.550
Model 2	0.36	(0.17, 0.77)	**0.008**	0.49	(0.22, 1.13)	0.095	0.33	(0.14, 0.75)	**0.008**	0.83	(0.49, 1.40)	0.474

Logistic regression analysis, with Model 1 as univariate analysis without adjustment; and Model 2 adjusted for covariates including: age, sex, height, weight, delivery way, household income, parental education, physical activity, use of calcium and multi-vitamin supplements, dietary intake of energy, protein, fat, carbohydrate, cholesterol, calcium, and vitamin D.

By defining abdominal obesity as an age- and sex-specific abdominal FMP ≥ 85th percentile, the higher dietary intake of anthocyanidin was associated with a 41.0% lower risk (*OR*: 0.59; *95%CI*: 0.37, 0.94) of abdominal obesity in total subjects and a 64.0% lower risk (*OR*: 0.36; *95%CI*: 0.17, 0.77) of abdominal obesity in boys. Besides, the higher dietary intake of cyanidin was associated with a 44.0% lower risk (*OR*: 0.56; *95%CI*: 0.35, 0.90) of abdominal obesity in total subjects and a 67% lower risk (*OR*: 0.33; *95%CI*: 0.14, 0.75) of abdominal obesity in boys. No significant associations were observed between other dietary anthocyanidin exposures and abdominal obesity (Table 3). Significant protective association between anthocyanidin and handgrip strength was found after adjusted for several covariates (e.g., age, sex, height, and weight); however, the association vanished after further adjustment of other nutrients, including protein, fat, carbohydrate, cholesterol, calcium, and vitamin D (Supplementary Table 6).

## Discussion

In our study, we found that dietary anthocyanidin and its compounds tended to be associated with better body composition, but not handgrip strength in children. Besides, higher anthocyanidin and cyanidin were associated with lower risk of abdominal obesity.

Consistent with our results, the benefit influences of anthocyanidin in adiposity had been reported among adults in several studies. Supplement of anthocyanidin from rich black soybean extract leads to reduction in abdominal fat, inflammatory factors, and better lipid profiles in adults with higher BMI or waist circumference (WC) (16). Bilberry supplementation contributed to decreased weight and WC in overweight or obese women in another RCT study (19). Higher dietary anthocyanidin (per one *SD* increase, 10 mg/d) was associated with 0.10 kg decrease of weight in a large population of 1,24,086 US adults from three prospective cohorts followed up to 24 years (20). Although the supplementation of several berries does not change the body composition or weight in subjects with metabolism syndrome, better lipid profile improvements were observed after the supplementation (28–30), which was consistent with the results found in another observational study (21). Intervention using anthocyanidin sourced from black raspberry does not affect the development in obese mice models (31). The heterogeneity of berries or different bioactive dose of anthocyanidin might contribute to the inconsistent results in adults. Generally, our results together with most of the former evidences emphasized the bright prospect of anthocyanidin in obesity prevention; however, well-designed prospective epidemiological studies are urgently needed in children.

In our study, anthocyanidin and its components were majorly sourced from vegetables and fruits, and they were positively correlated (*r’* = 0.330–0.781, all *P* < 0.001). Therefore, the protective associations of anthocyanidin on body composition might partly source from the benefit of vegetables and fruits as reported previously (32), and the associations were not adjusted for dietary intake of fruits and vegetables in our study in case of over adjustments. Several nutrients (fat, protein, carbohydrate, cholesterol, calcium, and vitamin D) were found to be associated with body composition or obesity before (33–36). In our study, similar positive relationships with anthocyanidins were observed for the dietary intake of calcium (partly source from fruits and vegetables) and carbohydrate (*r’* = 0.102–0.202, *P* < 0.05). We found that calcium was correlated with fruits and vegetables (*r’* = 0.184 and 0.186, *P* < 0.05), and carbohydrate was correlated with fruits (*r’* = 0.141, *P =* 0.003) in our study (data not shown), which might partly explain the relationships between anthocyanidin and these nutrients. No significant relationships were discovered between anthocyanidin and other nutrients, including protein, fat, cholesterol, and vitamin D, which suggested that the associations of anthocyanidin with body compositions did not generate from the decreased intake of fat and cholesterol (detrimental for body composition) or the increased intake of protein and vitamin D (favorable for body composition). Nevertheless, the associations between anthocyanidin and body composition were robust even after the adjustment of these nutrients, which suggested that the associations were not biased by these nutrients.

The dietary intake of anthocyanidin ranged from 0.50 to 27.7 mg/d, with a median (range interquartile) of 6.88 (4.06, 9.01) mg/d in our study. The amount was slightly lower than those observed in a study based on three large cohorts of US adults (median: 8.0–8.3 mg/d, range: 2.0–24.3 mg/d) (37). For calculating the total amount of anthocyanidin, only three components were included (delphinidin, cyanidin, and peonidin). Three other major components in foods (malvidin, pelargonidin, and pelargonidin) were not included because the database was not available in the latest Food Composition in China (25). Therefore, the total mounts of dietary anthocyanidin might be underestimated in our study; however, this might lead to an underestimation of the protective associations between anthocyanidin and body compositions, instead of overestimated associations.

Handgrip strength was positively correlated with LM (*r’* = 0.101 to 0.827, *P* < 0.05), but negatively correlated with FM (*r’* = −0.172 to −0.282, *P* < 0.01) and FMP (*r’* = −0.123 to −0.209, *P* < 0.01) after adjusted for age, sex, height, and weight (data not shown). However, we failed to observe the significant associations between anthocyanidins and handgrip strength. In fact, significant protective association between anthocyanidin and handgrip strength was found after adjusted for several covariates; however, the association vanished after further adjustments of other nutrients. Therefore, the associations between anthocyanidins and handgrip strength might be biased by other nutrients. The absence of several anthocyanidin components might lead to the underestimate of the amount of anthocyanidin and its protective influences. Besides, it could be possible that the influences of anthocyanidin on body composition are not quantitative enough to change the qualitative functions (handgrip strength). More studies were needed for the examination of our results.

Several mechanisms might contribute to the benefits of anthocyanin/anthocyanidin (A/A) against obesity (38). First, A/A contribute to an increase in the circulatory antioxidant status and capacity, lowering the oxidative stress (39, 40), which plays a major role in the development of obesity (41). Second, A/A might prevent obesity by confronting the influences of inflammatory effects through the NF-κB pathways (42) and by lowering the production of lipopolysaccharide-induced NO release and inducible nitric oxide synthase and the expression of cyclooxygenases-2 (43). Third, A/A might regulate the expression of adipocyte-specific gene by increasing the activity of AMP-activated protein kinase and subsequent reactions (44). Besides, the influences of A/A on gut microbiota might also contribute to obesity control (45).

There are several advantages of our study. First, to the best of our knowledge, this is the first study exploring the associations of anthocyanidin with body composition in children. Second, we used body composition measured by the gold standard as outcomes at multi-sites, which provided more precise information of fat distributions. Third, outcomes of abdominal obesity and handgrip strength were also included in the study (although no significant associations were observed), which provided further information in these fields. Finally, a large series of potential covariates were controlled in the analyses; therefore, we might largely avoid potential confounding from these factors. There are several limitations that merit careful consideration. First, the design of the study was cross-sectional, which only showed association instead of causality. Second, data of several other compounds of anthocyanidin (e.g., malvidin, pelargonidin, and pelargonidin) were unavailable in the latest Chinese Food Composition (25). Therefore, we could not analyze the associations of the rest of anthocyanidin compounds. However, this might underestimate, instead of overestimate, the protective associations of anthocyanidin. Third, children included in the study had a relatively narrow age range; therefore, the results might not be well extrapolated to children in other age groups. Further studies with prospective design and large age range were needed for the examination of our study. Finally, subjects were recruited as volunteers through letters or advertisement instead of random sampling. The response rate of the recruitment was low, because children and their guardians could choose to participate or not in the study (as volunteers). These might attenuate the representative of our study. We controlled a series of potential covariates to attenuate the influences. Besides, the association between anthocyanidin and abdominal obesity was not modified by all the covariates included in the analyses, including age, sex, economic status, household income, parental education, delivery way, use of calcium and multi-vitamin supplements, physical activities, and dietary intake of several other nutrients (*P*_-interaction_ = 0.056–0.977). Therefore, the associations of anthocyanidins and body composition might be able to extrapolate the subjects with different situations of these variables.

## Conclusion

In our study, higher dietary intake of anthocyanidin and its compounds tended to be associated with higher LM, lower FM, and lower FMP in children at early age. More prospective studies were needed for further examination of the associations.

## Supplementary Material

Associations of dietary anthocyanidins intake with body composition in Chinese children: a cross-sectional study
