# Supplementary material for: Associations of dietary anthocyanidins intake with body composition in Chinese children: a cross-sectional study
Source: Food Nutr Res. 2021 Aug 4;65:10.29219/fnr.v65.4428. doi: 10.29219/fnr.v65.4428 (PMC8344405; doi:10.29219/fnr.v65.4428)
Supplement: Associations of dietary anthocyanidins intake with body composition in Chinese children: a cross-sectional study [file FNR-65-4428-s001.docx]

**Supplemental Table 1: Comparison of the dietary anthocyanidins intake by the quartile of the body composition at multi-sites.**

|  | Quartile of body composition | | | |  |
| --- | --- | --- | --- | --- | --- |
|  | Q1 | Q2 | Q3 | Q4 | P-value ^a^ |
| Whole body FM |  |  |  |  |  |
| Anthocyanidin, mg/d ^b^ | 7.36 ± 3.99 | 7.16 ± 4.52 | 6.90 ± 3.82 | 6.09 ± 3.83 | 0.096 |
| Delphinidin, mg/d ^b^ | 0.67 ± 0.49 | 0.59 ± 0.44 | 0.54 ± 0.39 | **0.50 ± 0.39^*^** | **0.019** |
| Cyanidin, mg/d ^b^ | 5.80 ± 3.97 | 5.62 ± 3.97 | 5.37 ± 3.32 | 4.65 ± 3.42 | 0.082 |
| Peonidin, mg/d ^b^ | 0.93 ± 0.58 | 0.96 ± 0.82 | 0.96 ± 0.70 | 0.92 ± 0.78 | 0.963 |
| Whole body LM |  |  |  |  |  |
| Anthocyanidin, mg/d ^b^ | 6.73 ± 3.90 | 7.02 ± 4.20 | 6.65 ± 3.89 | 7.11 ± 4.29 | 0.796 |
| Delphinidin, mg/d ^b^ | 0.58 ± 0.36 | 0.61 ± 0.53 | 0.52 ± 0.33 | 0.60 ± 0.50 | 0.369 |
| Cyanidin, mg/d ^b^ | 5.89 ± 3.57 | 5.44 ± 3.60 | 5.23 ± 3.55 | 5.48 ± 3.74 | 0.944 |
| Peonidin, mg/d ^b^ | 0.90 ± 0.63 | 0.98 ± 0.77 | 0.91 ± 0.78 | 0.99 ± 0.71 | 0.709 |
| Whole body FMP |  |  |  |  |  |
| Anthocyanidin, mg/d ^b^ | 7.56 ± 4.01 | 7.02 ± 4.05 | 6.97 ± 4.44 | **5.96 ± 3.62^*^** | **0.027** |
| Delphinidin, mg/d ^b^ | 0.66 ± 0.49 | 0.63 ± 0.44 | **0.50 ± 0.38^*^** | 0.52 ± 4.05 | **0.008** |
| Cyanidin, mg/d ^b^ | 5.98 ± 3.45 | 5.42 ± 3.80 | 5.52 ± 3.92 | **4.51 ± 3.09^*^** | **0.019** |
| Peonidin, mg/d ^b^ | 0.94 ± 0.61 | 0.98 ± 0.73 | 0.95 ± 0.82 | 0.91 ± 0.72 | 0.922 |
| Trunk FM |  |  |  |  |  |
| Anthocyanidin, mg/d ^b^ | 7.72 ± 4.30 | 6.96 ± 4.25 | 6.59 ± 3.60 | **6.24 ± 3.98^*^** | **0.040** |
| Delphinidin, mg/d ^b^ | 0.68 ± 0.48 | 0.58 ± 0.43 | 0.53 ± 0.41 | **0.52 ± 0.40^*^** | **0.018** |
| Cyanidin, mg/d ^b^ | 6.15 ± 3.97 | 5.38 ± 3.62 | 5.16 ± 3.18 | **4.75 ± 3.52^*^** | **0.029** |
| Peonidin, mg/d ^b^ | 0.95 ± 0.61 | 1.00 ± 0.93 | 0.87 ± 0.49 | 0.95 ± 0.79 | 0.587 |
| Trunk LM |  |  |  |  |  |
| Anthocyanidin, mg/d ^b^ | 6.84 ± 4.35 | 7.17 ± 3.87 | 6.24 ± 3.74 | 7.25 ± 4.24 | 0.221 |
| Delphinidin, mg/d ^b^ | 0.59 ± 0.41 | 0.63 ± 0.48 | 0.49 ± 0.33 | 0.60 ± 0.49 | 0.113 |
| Cyanidin, mg/d ^b^ | 5.40 ± 3.86 | 5.60 ± 3.44 | 4.83 ± 3.36 | 5.60 ± 3.74 | 0.326 |
| Peonidin, mg/d ^b^ | 0.89 ± 0.62 | 0.96 ± 0.64 | 0.90 ± 0.80 | 1.02 ± 0.81 | 0.514 |
| Trunk FMP |  |  |  |  |  |
| Anthocyanidin, mg/d ^b^ | 7.66 ± 4.34 | 7.20 ± 3.67 | 6.65 ± 4.38 | **6.00 ± 3.68^*^** | **0.015** |
| Delphinidin, mg/d ^b^ | 0.65 ± 0.50 | 0.63 ± 0.42 | **0.50 ± 0.42^*^** | 0.52 ± 0.38 | **0.012** |
| Cyanidin, mg/d ^b^ | 6.11 ± 3.83 | 5.55 ± 3.40 | 5.23 ± 3.88 | **4.54 ± 3.13^**^** | **0.010** |
| Peonidin, mg/d ^b^ | 0.93 ± 0.63 | 1.02 ± 0.84 | 0.90 ± 0.66 | 0.93 ± 0.75 | 0.585 |
| Limbs FM |  |  |  |  |  |
| Anthocyanidin, mg/d ^b^ | 7.37 ± 3.95 | 6.91 ± 4.29 | 7.02 ± 4.13 | 6.21 ± 3.84 | 0.180 |
| Delphinidin, mg/d ^b^ | 0.65 ± 0.46 | 0.61 ± 0.47 | 0.55 ± 0.39 | 0.51 ± 0.40 | 0.069 |
| Cyanidin, mg/d ^b^ | 5.83 ± 3.63 | 5.32 ± 3.63 | 5.55 ± 3.67 | 4.73 ± 3.45 | 0.125 |
| Peonidin, mg/d ^b^ | 0.93 ± 0.59 | 0.99 ± 0.82 | 0.91 ± 0.68 | 0.95 ± 0.78 | 0.860 |
| Limbs LM |  |  |  |  |  |
| Anthocyanidin, mg/d ^b^ | 7.03 ± 4.02 | 6.49 ± 3.95 | 6.86 ± 3.99 | 7.13 ± 4.30 | 0.659 |
| Delphinidin, mg/d ^b^ | 0.58 ± 0.43 | 0.57 ± 0.44 | 0.55 ± 0.37 | 0.60 ± 0.50 | 0.840 |
| Cyanidin, mg/d ^b^ | 5.56 ± 3.67 | 5.05 ± 3.43 | 5.34 ± 3.59 | 5.48 ± 3.76 | 0.719 |
| Peonidin, mg/d ^b^ | 0.92 ± 0.63 | 0.89 ± 0.62 | 0.97 ± 0.91 | 1.00 ± 0.70 | 0.696 |
| Limbs FMP |  |  |  |  |  |
| Anthocyanidin, mg/d ^b^ | 7.33 ± 3.86 | 7.48 ± 4.62 | 6.64 ± 3.92 | 6.06 ± 3.69 | **0.031** |
| Delphinidin, mg/d ^b^ | 0.67 ± 0.52 | 0.63 ± 0.46 | **0.48 ± 0.28^**^** | 0.53 ± 0.41 | **0.003** |
| Cyanidin, mg/d ^b^ | 5.75 ± 3.30 | 5.87 ± 4.24 | 5.24 ± 3.54 | **4.57 ± 3.16^¶^** | **0.026** |
| Peonidin, mg/d ^b^ | 0.92 ± 0.60 | 1.00 ± 0.77 | 0.91 ± 0.78 | 0.94 ± 0.73 | 0.798 |
| Android area FM |  |  |  |  |  |
| Anthocyanidin, mg/d ^b^ | 7.87 ± 4.73 | 6.65 ± 3.86 | 6.62 ± 3.61 | **6.37 ± 3.86^*^** | **0.026** |
| Delphinidin, mg/d ^b^ | 0.70 ± 0.52 | 0.56 ± 0.43 | **0.53 ± 0.36^*^** | **0.51 ± 0.40^**^** | **0.005** |
| Cyanidin, mg/d ^b^ | 6.25 ± 4.27 | 5.13 ± 3.41 | 5.18 ± 3.08 | **4.87 ± 3.45^*^** | **0.021** |
| Peonidin, mg/d ^b^ | 0.96 ± 0.64 | 0.97 ± 0.86 | 0.88 ± 0.60 | 0.96 ± 0.78 | 0.793 |
| Android area LM |  |  |  |  |  |
| Anthocyanidin, mg/d ^b^ | 6.92 ± 4.27 | 6.82 ± 3.93 | 6.82 ± 3.85 | 6.94 ± 4.25 | 0.994 |
| Delphinidin, mg/d ^b^ | 0.61 ± 0.43 | 0.59 ± 0.47 | 0.53 ± 0.35 | 0.58 ± 0.48 | 0.516 |
| Cyanidin, mg/d ^b^ | 5.43 ± 3.76 | 5.27 ± 3.46 | 5.36 ± 3.54 | 5.37 ± 3.71 | 0.990 |
| Peonidin, mg/d ^b^ | 0.91 ± 0.61 | 0.98 ± 0.79 | 0.94 ± 0.82 | 0.95 ± 0.67 | 0.939 |
| Android area FMP |  |  |  |  |  |
| Anthocyanidin, mg/d ^b^ | 7.74 ± 4.32 | 7.18 ± 4.26 | 6.51 ± 3.86 | **6.07 ± 3.62^*^** | **0.011** |
| Delphinidin, mg/d ^b^ | 0.71 ± 0.52 | 0.57 ± 0.43 | **0.48 ± 0.33^**^** | **0.55 ± 0.41^*^** | **0.001** |
| Cyanidin, mg/d ^b^ | 6.09 ± 3.82 | 5.65 ± 3.85 | 5.11 ± 3.49 | **4.58 ± 3.07^**^** | **0.010** |
| Peonidin, mg/d ^b^ | 0.96 ± 0.64 | 0.99 ± 0.87 | 0.90 ± 0.64 | 0.93 ± 0.73 | 0.788 |
| Gynoid area FM |  |  |  |  |  |
| Anthocyanidin, mg/d ^b^ | 7.40 ± 3.92 | 7.02 ± 4.56 | 6.74 ± 3.81 | 6.35 ± 3.91 | 0.257 |
| Delphinidin, mg/d ^b^ | 0.65 ± 0.48 | 0.61 ± 0.47 | 0.54 ± 0.38 | 0.50 ± 0.39 | 0.040 |
| Cyanidin, mg/d ^b^ | 5.86 ± 3.59 | 5.43 ± 3.98 | 5.23 ± 3.32 | 4.92 ± 3.48 | 0.255 |
| Peonidin, mg/d ^b^ | 0.94 ± 0.59 | 0.99 ± 0.82 | 0.94 ± 0.77 | 0.90 ± 0.71 | 0.835 |
| Gynoid area LM |  |  |  |  |  |
| Anthocyanidin, mg/d ^b^ | 7.15 ± 4.49 | 6.69 ± 3.67 | 6.58 ± 3.84 | 7.09 ± 4.22 | 0.649 |
| Delphinidin, mg/d ^b^ | 0.64 ± 0.51 | 0.54 ± 0.36 | 0.55 ± 0.37 | 0.58 ± 0.48 | 0.311 |
| Cyanidin, mg/d ^b^ | 5.63 ± 3.95 | 5.17 ± 3.27 | 5.11 ± 3.46 | 5.52 ± 3.73 | 0.640 |
| Peonidin, mg/d ^b^ | 0.92 ± 0.68 | 0.99 ± 0.84 | 0.90 ± 0.70 | 0.97 ± 0.66 | 0.792 |
| Gynoid area FMP |  |  |  |  |  |
| Anthocyanidin, mg/d ^b^ | 7.58 ± 4.05 | 7.06 ± 4.02 | 7.08 ± 4.35 | **5.79 ± 3.64^**^** | **0.007** |
| Delphinidin, mg/d ^b^ | 0.67 ± 0.52 | 0.61 ± 0.41 | 0.55 ± 0.46 | **0.47 ± 0.31^**^** | **0.005** |
| Cyanidin, mg/d ^b^ | 5.98 ± 3.50 | 5.43 ± 3.78 | 5.57 ± 3.76 | **4.46 ± 3.24^**^** | **0.012** |
| Peonidin, mg/d ^b^ | 0.95 ± 0.65 | 1.03 ± 0.88 | 0.95 ± 0.65 | 0.85 ± 0.69 | 0.287 |

One-way ANOVA analysis. ^a:^ P value of the differences between groups. ^b:^ dietary intakes of nutrients were energy adjusted. **^*:^** P<0.05, **^**:^** P<0.01 compared with Q1. **^¶:^** P<0.05, compared with Q1.

**Supplemental Table 2. Relationship between dietary anthocyanidins and other nutrients.**

|  | Anthocyanidin | | Delphinidin | | Cyanidin | | Peonidin | |
| --- | --- | --- | --- | --- | --- | --- | --- | --- |
|  | *r’* | *P* | *r’* | *P* | *r’* | *P* | *r’* | *P* |
| Protein | 0.045 | 0.340 | 0.088 | 0.063 | 0.034 | 0.472 | 0.060 | 0.202 |
| Fat | -0.087 | 0.066 | -0.079 | 0.095 | -0.076 | 0.108 | -0.028 | 0.549 |
| Carbohydrate | 0.116 | **0.014** | 0.108 | **0.022** | 0.102 | **0.030** | 0.056 | 0.234 |
| Cholesterol | 0.065 | 0.168 | 0.068 | 0.150 | 0.078 | 0.101 | 0.016 | 0.743 |
| Calcium | 0.176 | **<0.001** | 0.202 | **<0.001** | 0.173 | **<0.001** | 0.149 | **0.002** |
| Vitamin D | 0.011 | 0.823 | 0.005 | 0.913 | 0.019 | 0.686 | 0.013 | 0.782 |
| Vegetables | 0.404 | **<0.001** | 0.515 | **<0.001** | 0.330 | **<0.001** | 0.351 | **<0.001** |
| Fruits | 0.744 | **<0.001** | 0.397 | **<0.001** | 0.781 | **<0.001** | 0.326 | **<0.001** |

All the nutrients and foods were energy-adjusted using the residual method. Partial correlation analysis, adjusted for age and sex; All variables were log-transformed.

**Supplemental Table 3. Univariate analysis of associations between dietary anthocyanidins and body composition.**

|  | Anthocyanidin | | |  | Delphinidin | | |  | Cyanidin | | |  | Peonidin | | |
| --- | --- | --- | --- | --- | --- | --- | --- | --- | --- | --- | --- | --- | --- | --- | --- |
|  | *β* | *SE* | *P* |  | *β* | *SE* | *P* |  | *β* | *SE* | *P* |  | *β* | *SE* | *P* |
| Whole body | | | | | | | | | | | | | | | |
| FM, kg | -0.371 | 0.173 | **0.032** |  | -0.413 | 0.173 | **0.017** |  | -0.407 | 0.172 | **0.019** |  | 0.064 | 0.173 | 0.710 |
| LM, kg | 0.135 | 0.179 | 0.450 |  | -0.031 | 0.179 | 0.864 |  | 0.076 | 0.179 | 0.670 |  | 0.245 | 0.178 | 0.171 |
| FMP, % | -1.032 | 0.289 | **<0.001** |  | -1.047 | 0.289 | **<0.001** |  | -1.029 | 0.289 | **<0.001** |  | -0.208 | 0.293 | 0.478 |
| Trunk |  |  |  |  |  |  |  |  |  |  |  |  |  |  |  |
| FM, kg | -0.174 | 0.078 | **0.027** |  | -0.170 | 0.078 | **0.031** |  | -0.193 | 0.078 | **0.014** |  | 0.035 | 0.079 | 0.659 |
| LM, kg | 0.047 | 0.084 | 0.576 |  | -0.044 | 0.084 | 0.600 |  | 0.020 | 0.084 | 0.813 |  | 0.115 | 0.084 | 0.171 |
| FMP, % | -1.136 | 0.313 | **<0.001** |  | -0.966 | 0.314 | **0.002** |  | -1.154 | 0.312 | **<0.001** |  | -0.218 | 0.317 | 0.491 |
| Limbs | | | | | | | | | | | | | | | |
| FM, kg | -0.196 | 0.092 | **0.035** |  | -0.236 | 0.092 | **0.011** |  | -0.211 | 0.092 | **0.023** |  | 0.026 | 0.093 | 0.777 |
| LM, kg | 0.087 | 0.085 | 0.311 |  | 0.031 | 0.086 | 0.717 |  | 0.056 | 0.085 | 0.516 |  | 0.121 | 0.085 | 0.156 |
| FMP, % | -1.221 | 0.355 | **0.001** |  | -1.433 | 0.354 | **<0.001** |  | -1.188 | 0.356 | **0.001** |  | -0.284 | 0.360 | 0.430 |
| Android area | | | | | | | | | | | | | | | |
| FM, kg | -0.028 | 0.013 | **0.035** |  | -0.027 | 0.013 | **0.042** |  | -0.031 | 0.013 | **0.020** |  | 0.006 | 0.013 | 0.664 |
| LM, kg | 0.004 | 0.014 | 0.773 |  | -0.010 | 0.014 | 0.472 |  | 0.001 | 0.014 | 0.923 |  | 0.011 | 0.014 | 0.434 |
| FMP, % | -1.229 | 0.328 | **<0.001** |  | -1.048 | 0.330 | **0.002** |  | -1.260 | 0.327 | **<0.001** |  | -0.153 | 0.333 | 0.647 |
| Gynoid area | | | | | | | | | | | | | | | |
| FM, kg | -0.057 | 0.028 | **0.044** |  | -0.077 | 0.028 | **0.006** |  | -0.057 | 0.028 | **0.041** |  | -0.006 | 0.028 | 0.825 |
| LM, kg | 0.012 | 0.033 | 0.707 |  | -0.024 | 0.033 | 0.465 |  | 0.006 | 0.033 | 0.860 |  | 0.029 | 0.033 | 0.375 |
| FMP, % | -1.049 | 0.292 | **<0.001** |  | -1.162 | 0.292 | **<0.001** |  | -0.991 | 0.292 | **0.001** |  | -0.394 | 0.296 | 0.184 |

FM: fat mass; LM: lean mass; FMP: fat mass percentage;

Linear regression analysis without adjustment.

**Supplemental Table 4. Univariate analysis of associations between dietary anthocyanidins and body composition stratified by sex.**

|  | | Anthocyanidin | | |  | Delphinidin | | |  | Cyanidin | | |  | Peonidin | | |
| --- | --- | --- | --- | --- | --- | --- | --- | --- | --- | --- | --- | --- | --- | --- | --- | --- |
|  |  | *β* | *SE* | *P* |  | *β* | *SE* | *P* |  | *β* | *SE* | *P* |  | *β* | *SE* | *P* |
| Girls (N=197) | | | | | | | | | | | | | | | | |
| Whole body | FM, kg | -0.321 | 0.212 | 0.131 |  | -0.461 | 0.210 | **0.030** |  | -0.271 | 0.212 | 0.203 |  | -0.221 | 0.212 | 0.299 |
|  | LM, kg | 0.169 | 0.221 | 0.446 |  | -0.039 | 0.221 | 0.859 |  | 0.175 | 0.221 | 0.430 |  | 0.102 | 0.221 | 0.645 |
|  | FMP, % | -0.966 | 0.415 | **0.021** |  | -1.164 | 0.412 | **0.005** |  | -0.829 | 0.416 | **0.048** |  | -0.707 | 0.417 | 0.092 |
| Trunk | FM, kg | -0.141 | 0.096 | 0.143 |  | -0.192 | 0.095 | **0.045** |  | -0.122 | 0.096 | 0.203 |  | -0.084 | 0.096 | 0.381 |
|  | LM, kg | 0.047 | 0.106 | 0.660 |  | -0.071 | 0.106 | 0.504 |  | 0.054 | 0.106 | 0.614 |  | 0.037 | 0.106 | 0.727 |
|  | FMP, % | -0.965 | 0.460 | **0.037** |  | -1.035 | 0.459 | **0.025** |  | -0.852 | 0.461 | 0.066 |  | -0.673 | 0.462 | 0.147 |
| Limbs | FM, kg | -0.180 | 0.115 | 0.119 |  | -0.262 | 0.114 | **0.023** |  | -0.148 | 0.115 | 0.200 |  | -0.138 | 0.115 | 0.232 |
|  | LM, kg | 0.118 | 0.105 | 0.261 |  | 0.050 | 0.105 | 0.635 |  | 0.116 | 0.105 | 0.271 |  | 0.060 | 0.105 | 0.570 |
|  | FMP, % | -1.236 | 0.502 | **0.015** |  | -1.625 | 0.496 | **0.001** |  | -1.035 | 0.504 | **0.041** |  | -0.945 | 0.505 | 0.063 |
| Android area | FM, kg | -0.020 | 0.015 | 0.200 |  | -0.028 | 0.015 | 0.068 |  | -0.016 | 0.015 | 0.279 |  | -0.013 | 0.015 | 0.383 |
|  | LM, kg | 0.004 | 0.017 | 0.800 |  | -0.023 | 0.017 | 0.189 |  | 0.009 | 0.017 | 0.607 |  | -0.007 | 0.017 | 0.689 |
|  | FMP, % | -0.996 | 0.459 | **0.031** |  | -0.914 | 0.460 | **0.048** |  | -0.931 | 0.459 | **0.044** |  | -0.514 | 0.463 | 0.268 |
| Gynoid area | FM, kg | -0.047 | 0.036 | 0.191 |  | -0.082 | 0.036 | **0.022** |  | -0.034 | 0.036 | 0.343 |  | -0.053 | 0.036 | 0.146 |
|  | LM, kg | 0.016 | 0.041 | 0.689 |  | -0.028 | 0.041 | 0.495 |  | 0.022 | 0.041 | 0.594 |  | -0.001 | 0.041 | 0.981 |
|  | FMP, % | -0.911 | 0.408 | **0.027** |  | -1.188 | 0.404 | **0.004** |  | -0.722 | 0.410 | 0.080 |  | -0.915 | 0.408 | **0.026** |
| Boys (N=255) | | | | | | | | | | | | | | | | |
| Whole body | FM, kg | -0.410 | 0.259 | 0.115 |  | -0.376 | 0.260 | 0.149 |  | -0.511 | 0.258 | **0.049** |  | 0.285 | 0.260 | 0.274 |
|  | LM, kg | 0.109 | 0.255 | 0.669 |  | -0.024 | 0.256 | 0.926 |  | 0.000 | 0.255 | 1.000 |  | 0.355 | 0.254 | 0.164 |
|  | FMP, % | -1.083 | 0.390 | **0.006** |  | -0.957 | 0.393 | **0.016** |  | -1.183 | 0.389 | **0.003** |  | 0.177 | 0.396 | 0.655 |
| Trunk | FM, kg | -0.199 | 0.118 | 0.092 |  | -0.154 | 0.118 | 0.196 |  | -0.247 | 0.117 | **0.036** |  | 0.127 | 0.118 | 0.285 |
|  | LM, kg | 0.047 | 0.119 | 0.694 |  | -0.023 | 0.120 | 0.846 |  | -0.006 | 0.119 | 0.959 |  | 0.175 | 0.119 | 0.142 |
|  | FMP, % | -1.268 | 0.420 | **0.003** |  | -0.912 | 0.424 | **0.032** |  | -1.388 | 0.418 | **0.001** |  | 0.133 | 0.427 | 0.757 |
| Limbs | FM, kg | -0.208 | 0.138 | 0.133 |  | -0.216 | 0.139 | 0.119 |  | -0.260 | 0.138 | 0.060 |  | 0.153 | 0.138 | 0.269 |
|  | LM, kg | 0.062 | 0.123 | 0.612 |  | 0.016 | 0.123 | 0.894 |  | 0.009 | 0.123 | 0.942 |  | 0.169 | 0.122 | 0.169 |
|  | FMP, % | -1.210 | 0.478 | **0.012** |  | -1.285 | 0.479 | **0.008** |  | -1.306 | 0.477 | **0.007** |  | 0.227 | 0.484 | 0.640 |
| Android area | FM, kg | -0.035 | 0.020 | 0.089 |  | -0.027 | 0.021 | 0.194 |  | -0.043 | 0.020 | **0.038** |  | 0.021 | 0.020 | 0.316 |
|  | LM, kg | 0.004 | 0.021 | 0.849 |  | -0.001 | 0.021 | 0.970 |  | -0.004 | 0.021 | 0.831 |  | 0.025 | 0.021 | 0.224 |
|  | FMP, % | -1.409 | 0.459 | **0.002** |  | -1.152 | 0.463 | **0.013** |  | -1.514 | 0.458 | **0.001** |  | 0.126 | 0.467 | 0.787 |
| Gynoid area | FM, kg | -0.064 | 0.041 | 0.122 |  | -0.073 | 0.041 | 0.078 |  | -0.075 | 0.041 | 0.069 |  | 0.030 | 0.041 | 0.474 |
|  | LM, kg | 0.009 | 0.048 | 0.844 |  | -0.022 | 0.049 | 0.657 |  | -0.006 | 0.048 | 0.895 |  | 0.053 | 0.048 | 0.273 |
|  | FMP, % | -1.156 | 0.380 | **0.003** |  | -1.141 | 0.382 | **0.003** |  | -1.199 | 0.380 | **0.002** |  | 0.009 | 0.387 | 0.982 |

FM: fat mass; LM: lean mass; FMP: fat mass percentage;

Linear regression analysis without adjustment.

**Supplemental Table 5. Associations of dietary anthocyanidins with body composition after adjusted for potential covariates stratified by sex.**

|  | | Anthocyanidin | | |  | Delphinidin | | |  | Cyanidin | | |  | Peonidin | | |
| --- | --- | --- | --- | --- | --- | --- | --- | --- | --- | --- | --- | --- | --- | --- | --- | --- |
|  |  | *β* | *SE* | *P* |  | *β* | *SE* | *P* |  | *β* | *SE* | *P* |  | *β* | *SE* | *P* |
| Girls (N=197) | |  |  |  |  |  |  |  |  |  |  |  |  |  |  |  |
| Whole body | FM, kg | -0.127 | 0.077 | 0.100 |  | -0.130 | 0.076 | 0.089 |  | -0.117 | 0.076 | 0.128 |  | -0.051 | 0.074 | 0.489 |
|  | LM, kg | 0.178 | 0.076 | **0.020** |  | 0.154 | 0.076 | **0.044** |  | 0.166 | 0.076 | **0.030** |  | 0.080 | 0.074 | 0.280 |
|  | FMP, % | -0.535 | 0.289 | 0.066 |  | -0.597 | 0.287 | **0.039** |  | -0.473 | 0.289 | 0.103 |  | -0.291 | 0.278 | 0.298 |
| Trunk | FM, kg | -0.051 | 0.038 | 0.187 |  | -0.041 | 0.038 | 0.285 |  | -0.052 | 0.038 | 0.180 |  | -0.005 | 0.037 | 0.899 |
|  | LM, kg | 0.069 | 0.041 | 0.089 |  | 0.033 | 0.041 | 0.416 |  | 0.067 | 0.040 | 0.099 |  | 0.036 | 0.039 | 0.360 |
|  | FMP, % | -0.533 | 0.320 | 0.098 |  | -0.423 | 0.319 | 0.186 |  | -0.503 | 0.319 | 0.116 |  | -0.230 | 0.308 | 0.455 |
| Limbs | FM, kg | -0.077 | 0.046 | 0.093 |  | -0.088 | 0.045 | 0.053 |  | -0.066 | 0.045 | 0.149 |  | -0.050 | 0.044 | 0.254 |
|  | LM, kg | 0.102 | 0.044 | **0.020** |  | 0.124 | 0.043 | **0.004** |  | 0.094 | 0.044 | **0.033** |  | 0.034 | 0.042 | 0.419 |
|  | FMP, % | -0.694 | 0.387 | 0.074 |  | -0.977 | 0.381 | **0.011** |  | -0.575 | 0.386 | 0.139 |  | -0.443 | 0.372 | 0.235 |
| Android area | FM, kg | -0.005 | 0.006 | 0.370 |  | -0.003 | 0.006 | 0.607 |  | -0.006 | 0.006 | 0.356 |  | -0.001 | 0.006 | 0.878 |
|  | LM, kg | 0.012 | 0.009 | 0.177 |  | -0.001 | 0.009 | 0.943 |  | 0.014 | 0.009 | 0.107 |  | -0.003 | 0.009 | 0.765 |
|  | FMP, % | -0.620 | 0.311 | **0.048** |  | -0.303 | 0.312 | 0.333 |  | -0.644 | 0.309 | **0.039** |  | -0.101 | 0.301 | 0.738 |
| Gynoid area | FM, kg | -0.017 | 0.015 | 0.234 |  | -0.029 | 0.014 | **0.043** |  | -0.010 | 0.014 | 0.479 |  | -0.027 | 0.014 | **0.049** |
|  | LM, kg | 0.030 | 0.017 | 0.082 |  | 0.014 | 0.017 | 0.400 |  | 0.031 | 0.017 | 0.067 |  | 0.004 | 0.016 | 0.820 |
|  | FMP, % | -0.578 | 0.339 | 0.090 |  | -0.772 | 0.335 | **0.022** |  | -0.439 | 0.339 | 0.197 |  | -0.576 | 0.324 | 0.077 |
| Boys (N=255) | |  |  |  |  |  |  |  |  |  |  |  |  |  |  |  |
| Whole body | FM, kg | -0.159 | 0.077 | **0.041** |  | -0.055 | 0.077 | 0.477 |  | -0.142 | 0.077 | 0.068 |  | -0.103 | 0.071 | 0.148 |
|  | LM, kg | 0.291 | 0.073 | **<0.001** |  | 0.147 | 0.074 | **0.049** |  | 0.285 | 0.073 | **<0.001** |  | 0.060 | 0.069 | 0.390 |
|  | FMP, % | -0.798 | 0.262 | **0.003** |  | -0.549 | 0.262 | **0.037** |  | -0.728 | 0.262 | **0.006** |  | -0.341 | 0.244 | 0.164 |
| Trunk | FM, kg | -0.086 | 0.038 | **0.025** |  | 0.001 | 0.038 | 0.985 |  | -0.082 | 0.038 | **0.032** |  | -0.047 | 0.035 | 0.179 |
|  | LM, kg | 0.139 | 0.036 | **<0.001** |  | 0.059 | 0.037 | 0.110 |  | 0.134 | 0.036 | **<0.001** |  | 0.038 | 0.034 | 0.261 |
|  | FMP, % | -0.973 | 0.266 | **<0.001** |  | -0.409 | 0.267 | 0.128 |  | -0.915 | 0.265 | **0.001** |  | -0.435 | 0.248 | 0.081 |
| Limbs | FM, kg | -0.072 | 0.048 | 0.130 |  | -0.051 | 0.047 | 0.278 |  | -0.060 | 0.047 | 0.206 |  | -0.052 | 0.044 | 0.231 |
|  | LM, kg | 0.151 | 0.043 | **0.001** |  | 0.104 | 0.043 | **0.017** |  | 0.144 | 0.043 | **0.001** |  | 0.034 | 0.041 | 0.397 |
|  | FMP, % | -0.892 | 0.377 | **0.019** |  | -0.899 | 0.372 | **0.016** |  | -0.788 | 0.375 | **0.037** |  | -0.350 | 0.348 | 0.316 |
| Android area | FM, kg | -0.013 | 0.007 | 0.087 |  | 0.002 | 0.007 | 0.822 |  | -0.012 | 0.007 | 0.112 |  | -0.008 | 0.007 | 0.238 |
|  | LM, kg | 0.017 | 0.008 | **0.047** |  | 0.013 | 0.008 | 0.123 |  | 0.017 | 0.008 | **0.042** |  | -0.001 | 0.008 | 0.904 |
|  | FMP, % | -0.986 | 0.305 | **0.001** |  | -0.547 | 0.305 | 0.074 |  | -0.911 | 0.304 | **0.003** |  | -0.425 | 0.284 | 0.136 |
| Gynoid area | FM, kg | -0.032 | 0.016 | **0.042** |  | -0.030 | 0.015 | 0.054 |  | -0.023 | 0.016 | 0.131 |  | -0.032 | 0.014 | **0.026** |
|  | LM, kg | 0.046 | 0.017 | **0.008** |  | 0.014 | 0.017 | 0.405 |  | 0.051 | 0.017 | **0.003** |  | -0.011 | 0.016 | 0.480 |
|  | FMP, % | -1.025 | 0.342 | **0.003** |  | -0.900 | 0.339 | **0.009** |  | -0.931 | 0.341 | **0.007** |  | -0.367 | 0.318 | 0.250 |

FM: fat mass; LM: lean mass; FMP: fat mass percentage;

Linear regression analysis, without adjustment for covariates including: age, sex, height, weight, delivery way, household income, parental education, physical activity, use of calcium and multi-vitamin supplements, dietary intake of energy, protein, fat, carbohydrate, cholesterol, calcium, vitamin D.

**Supplemental Table 6: Associations of dietary anthocyanidins with handgrip strength.**

| Handgrip strength, kg | Per SD increase of dietary anthocyanidin and its main compounds | | | | | | | | | | | | | | |
| --- | --- | --- | --- | --- | --- | --- | --- | --- | --- | --- | --- | --- | --- | --- | --- |
|  | Anthocyanidin | | |  | Delphinidin | | |  | Cyanidin | | |  | Peonidin | | |
|  | *β* | *se* | *p* |  | *β* | *se* | *p* |  | *β* | *se* | *p* |  | *β* | *se* | *p* |
| Total (N=452) | | | | | | | | | | | | | | | |
| Model 1 | 0.161 | 0.135 | 0.231 |  | 0.041 | 0.134 | 0.759 |  | 0.098 | 0.134 | 0.466 |  | 0.241 | 0.134 | 0.072 |
| Model 2 | 0.185 | 0.092 | **0.044** |  | 0.151 | 0.091 | 0.100 |  | 0.158 | 0.092 | 0.088 |  | 0.126 | 0.091 | 0.169 |
| Model 3 | 0.127 | 0.098 | 0.193 |  | 0.097 | 0.097 | 0.314 |  | 0.093 | 0.098 | 0.344 |  | 0.099 | 0.092 | 0.282 |
| Girls (N=197) | | | | | | | | | | | | | | | |
| Model 1 | 0.115 | 0.172 | 0.504 |  | 0.183 | 0.176 | 0.301 |  | 0.235 | 0.180 | 0.194 |  | 0.112 | 0.187 | 0.549 |
| Model 2 | 0.212 | 0.134 | 0.115 |  | 0.278 | 0.133 | **0.038** |  | 0.201 | 0.134 | 0.135 |  | 0.035 | 0.133 | 0.790 |
| Model 3 | 0.165 | 0.139 | 0.236 |  | 0.158 | 0.135 | 0.244 |  | 0.137 | 0.135 | 0.312 |  | 0.018 | 0.130 | 0.892 |
| Boys (N=255) | | | | | | | | | | | | | | | |
| Model 1 | 0.274 | 0.197 | 0.165 |  | -0.018 | 0.191 | 0.923 |  | 0.010 | 0.187 | 0.958 |  | 0.330 | 0.181 | 0.071 |
| Model 2 | 0.202 | 0.128 | 0.117 |  | 0.058 | 0.128 | 0.652 |  | 0.166 | 0.129 | 0.198 |  | 0.222 | 0.128 | 0.083 |
| Model 3 | 0.123 | 0.139 | 0.376 |  | -0.059 | 0.139 | 0.670 |  | 0.143 | 0.140 | 0.309 |  | 0.205 | 0.129 | 0.113 |

Logistic regression analysis, with Model 1 as univariate analysis without adjustment; Model 2 adjusted for covariates including: age, sex, height, weight, delivery way, household income, parental education, physical activity, use of calcium and multi-vitamin supplements, dietary intake of energy; and Model 3 further adjusted for dietary intake protein, fat, carbohydrate, cholesterol, calcium, vitamin D.
